# Neuropsychology of the temporal lobe: Luria's and contemporary conceptions

**DOI:** 10.1590/1980-57642018dn13-030001

**Published:** 2019

**Authors:** Tales Alexandre Aversi-Ferreira, Bruno Hideki Tamaishi-Watanabe, Micheli Patrícia de Fátima Magri, Roqueline A.G.M.F. Aversi-Ferreira

**Affiliations:** 1 Federal University of Alfenas Institute of Biomedical Sciences Department of Anatomy AlfenasMG Brazil Laboratory of Biomathematics and Physical Anthropology, Department of Anatomy, Institute of Biomedical Sciences, Federal University of Alfenas, Alfenas, MG, Brazil.; 2 Universidade Paulista Department of Health Nursing School São José do Rio PardoSP Brazil Nursing School, Department of Health, Universidade Paulista, São José do Rio Pardo, SP, Brazil.

**Keywords:** temporal lobe, neuropsychology, Luria, lobo temporal, neuropsicologia, Luria

## Abstract

Brain lesion studies currently employ techniques such as computed tomography, functional magnetic resonance imaging, single photon emission tomography and positron emission tomography. Famous neuropsychologist Alexander Romanovich Luria’s studies on cognition were conducted without the use of imaging technology for many years, in a large number of patients with brain lesions, and explored complex behavior and specific brain functions involving the lobes and subareas. For instance, he carried out several specific studies on memory and mental organization, reported in his books. The objective of this study is to associate recent studies in neuropsychology with Luria’s work specifically on the temporal lobe. According to the data studied, Luria’s epistemological foundation remains the basis for neuropsychological studies today, but new data on the temporal lobe in relation to epilepsy and hippocampus analysis have been introduced into the scope of neuropsychology. This study focuses on earlier data from Luria’s studies on the neuropsychological functions of the temporal lobe, comparing these with more recent data. However, in order to improve clinical aspects, a detailed study on the neuropsychological tests used for the temporal lobe should be performed.

## RESUMO

Atualmente, estudos de lesão cerebral implementam técnicas como tomografia computadorizada, ressonância magnética funcional, tomografia por emissão de fóton e tomografia por emissão de pósitrons. Estudos sobre cognição do neuropsicólogo Alexander Romanovich Luria foram realizados há vários anos sem o uso de tecnologia de imagem, com uma grande quantidade de pacientes com lesões cerebrais, envolvendo comportamento complexo e funções cerebrais específicas em relação aos lobos e suas subáreas. Por exemplo, ele realizou vários estudos sobre memória e organização mental mostrados em alguns de seus livros. O objetivo deste trabalho é associar estudos recentes em neuropsicologia com o trabalho de Luria sobre o lobo temporal, em específico. De acordo com os dados estudados aqui, a base epistemológica de Luria para os estudos neuropsicológicos atuais é ainda, fundamental, mas novos dados sobre o lobo temporal em relação à análise de epilepsia e hipocampo foram introduzidos no escopo da neuropsicologia. Neste trabalho foram priorizados os estudos das funções neuropsicológicas do lobo temporal dos dados anteriores dos estudos de Luria em relação aos mais recentes, no entanto, para fins de melhoria de aspectos clínicos, um estudo detalhado sobre os testes neuropsicológicos utilizados para o lobo temporal deveria ser realizado.

Alexander Romanovich Luria (1902-1977) performed several studies on the structures of the brain and behavior which contributed greatly to the philosophy of socio-cultural psychology. The associations observed between local brain lesions and behavioral changes led to the publication of his book, The Working Brain,^1^ which has since served as a foundation for the understanding of cerebral functions. In World War II, Luria conducted research in Kisegach on patients suffering from brain injuries and observed the possible relationship between these lesions and alterations in cognition and behavior.^2^^-^4

Through the use of a neuropsychological test, these findings, *inter alia*, made it possible to predict cognitive disorders based on the location of the injuries. This proved an important aspect in indicating the appropriate location for potential surgical intervention,^5^ particularly if the lesion occurred within the tertiary cortical areas of the brain which do not generate electrophysiological responses to stimuli - lesions which, unlike cases of stroke or inflammation, are undetectable.

However, brain lesion studies now implement other techniques such as Computed Tomography (CT), Functional Magnetic Resonance Imaging (FMRI), Single Photon Emission Tomography (SPECT) and Positron Emission Tomography (PET).^6^ Therefore, it is no longer necessary to wait until the patient dies in order to conduct a meticulous analysis of the brain's anatomy.

Luria's in-depth cognitive studies were carried out without the use of imaging technology for many years in a large number of patients with cerebral lesions,^1^^,^^7^^-^9 and explored specific complex behavior and brain functions involving the lobes and subareas, such as the parietal, frontal, occipital and temporal lobes, as well as the pre-frontal, temporal-parietal occipital, and pre-central lobules. For instance, he performed several specific studies on memory and mental organization reported in his books.^2^^,^^10^

According to Luria, his studies on behavior within the scope of psychology were also within the scope of sociocultural psychology formulated mainly by Vygotsky.^11^ However, for some years during World War II, Luria managed a hospital dedicated to caring for those wounded in battle, providing a great number of cases of patients with brain damage.^2^

In Luria's analysis of these patients, in which he applied the methods developed by himself and mainly Vygotsky, considering the mind to be a much more complex expression than the anatomical structure of the brain, i.e., using the philosophy of the sociocultural development of the mind,^11^ led to a very interesting interpretation of the brain's physiology and of the superior functions of the neocortex in modern humans.^9^^,^^12^

It was very important to reveal new insights in the cognitive interpretation of human behavior linked to local cerebral lesions, in contrast to the general tendency of psychiatrists to prioritize studying the cerebral lesions themselves.^13^ Oliver Sacks, in the preface of the book “The Man with a Shattered World: The History of a Brain Wound”,^2^ wrote that Luria's work was among the most important in neuroscience, conferring him the equivalent status of Freud in psychoanalysis, applied to the field of neuropsychology.^2^ Notwithstanding, Michael and Sheila Cole pointed out that few American psychologists know about Luria and his findings.^11^ The same holds true in Brazil, where the main psychologist studied is Vygotsky rather than Luria.^14^ However, Luria continued to further develop Vygotsky's perspective of the mind following his early death at age 37.

The objective of this paper is to review the main concepts Luria discussed on lesions and the neuropsychology of the temporal lobe (TL), responsible for hearing and other functions including memory and speech, and to compare them to modern insights gained from today's clinical, surgical and technological advancements such as imaging techniques. These comparisons of data can potentially improve clinicians' behavioral analysis of patients in general, as well as shed light on the path the field of neuropsychology has taken over recent years in relation to its original mission.

The general aspects of Luria's studies on the temporal lobe and new findings were analyzed without considering the more complex aspects of functions and structure. The main goal of this study is to associate recent studies in neurophysiology with Luria's work regarding the TL.

## METHODS

For the purposes of this manuscript, articles published since 1992 matching both of the key words *temporal lobe* and *neuropsychology* were searched. The search was performed using PubMed, Scopus, Medline, Elsevier, and Google Scholar.

Earlier articles and those focused on similar subjects were excluded from the study. Articles considered most appropriate for this paper's purpose were used. With regard to papers covering similar subjects, only the most recent were selected. For instance, 1078 papers on the subjects of TL and neuropsychology were found on PubMed, but only 65 met the exclusion criteria and were used subsequently included in this study.

**Figure 1:**
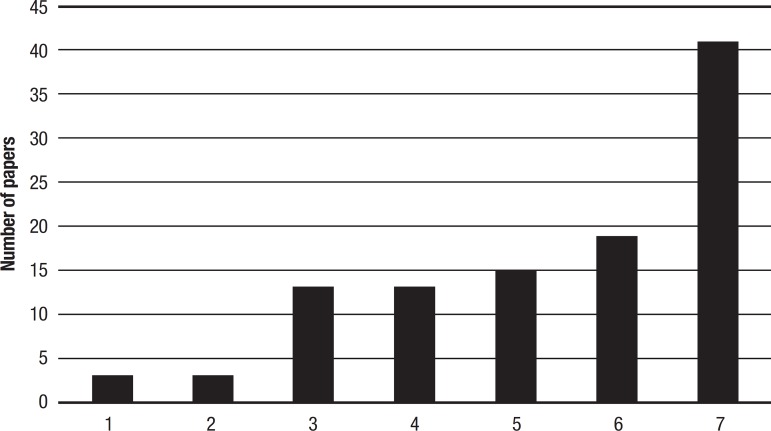
Number of papers for each subject studied for this manuscript after 1992. 1. neuropsychology and memory; 2. neuropsychology, epilepsy and memory; 3. neuropsychology; 4. neuropsychology and epilepsy; 5. epilepsy and memory; 6. memory; 7. epilepsy.

Regarding the selection of key words, these were separated into types: *neuropsychology* and *memory*; *neuropsychology, epilepsy* and *memory*; *neuropsychology*; *neuropsychology* and *epilepsy*; *epilepsy* and *memory*; *memory*; and *epilepsy* (Figure 1). The number of papers were counted considering the overlapping of these descriptors.

**Figure 2:**
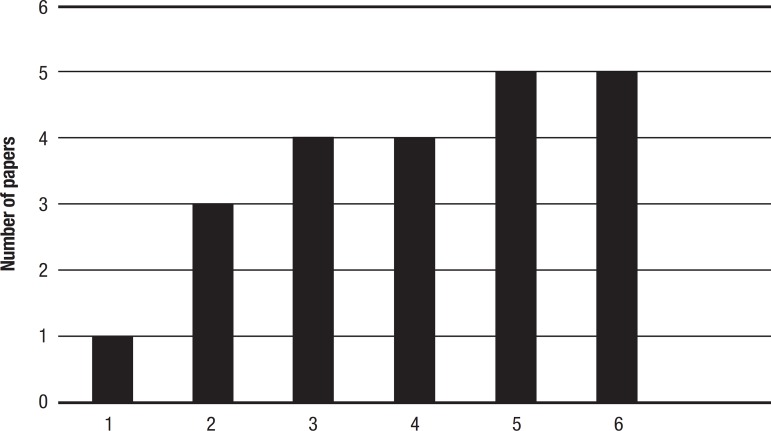
Number of papers by subject studied for this manuscript before Luria’s book “The Working Brain - Introduction to Neuropsychology”. 1. epilepsy; 2. aphasia; 3. neuropsychology; 4. memory; 5. cortical lesion; 6. hearing.

Luria's description of Neuropsychology was based on the book “The Working Brain - Introduction to the Neuropsychology” and the articles mentioned in it, which were therefore published before 1973. A total of 16 articles were selected (Figure 2), without considering the books or the overlapping of descriptors.

In order to compare the number of articles and their subjects, as well as the subjects from different eras, the Chi-squared test using StatPlus:mac AnalystSoft Inc. 2018 was applied.

The Chi-squared test is considered robust for small samples and non-parametric analysis. The expected data was obtained by dividing the number of subjects by 100%, considering that the subjects had the same probability of being studied.

Nevertheless, the number of samples was not always more than 5 for all cases, where the Chi-squared test is considered adequate for this kind of proportional analysis of discrete variables.

## RESULTS

The most common subject addressed in the recent papers analyzed in this manuscript was epilepsy, followed by memory and the association between epilepsy and memory (Figure 1). In earlier articles, the most common subject was hearing and cortical lesions, followed by memory and neuropsychology (Figure 2).

The Chi-square test indicated a significant difference [H_0_ accepted] among the subjects of recent papers of p<0.05, where the same occurred with the subjects of earlier papers for the same value of *p*. When comparing the subjects of recent and earlier papers, H_0_ was accepted, thereby indicating a significant difference for the group of subjects of *p*<0.05.

## DISCUSSION

### Luria's studies on the temporal lobe

Luria analyzed the functions of the temporal lobe, considering the pathologies and lesions of many patients,^1^ and wrote about their deficiencies and behavior based on his own observations and those reported in other articles.^15^^-^19 In his description of the temporal lobe, Luria cited the cytoarchitecture and physiology of the cortical areas that form the primary and secondary cortices widely recognized for the function of listening.^1^^,^^13^

In fact, in his initial description of the temporal lobe, Luria cited the excellence of the primary cortex of this region as an auditory area possessing somatotopical organization,^7^ such that the medial area is excited by high tones and the lateral by low tones.

The secondary area was cited as having acousticgnostic functions, including disturbances in auditory speech,^7^^,^^20^ in general terms.

According to Luria,^1^ projections in the auditory cortex reach both sides of each ear in the primary sensory zones, an impeding factor in the generation of complete deafness due to lesions of only one side of the temporal cortex. These projections involving the cortex prolong and stabilize auditory information.

The secondary zones of the temporal cortex [area 22 and part of Brodmann's area 21] have similar cytoarchitectural structures to the other secondary zones of the brain abundant in neurons with short axons and well-developed layers II and III, known as the external granular and external pyramidal layers, respectively,^7^ which maintain the specific modality for sensory function.

As summarized by Luria, in earlier studies on animals, secondary zone lesions caused a decrease in ability to combine different acoustic reflexes^15^^,^^21^ and, in humans, similar lesions to the left cortex superior temporal gyrus, impede individuals' capacity to differentiate between different combinations of sounds,^22^ as well as different rhythms.

The secondary zone of the left temporal cortex is responsible for the function of the analysis and synthesis of the sound of speech or qualified speech hearing, and forms part of the cerebral organization of articulated speech via the arcuate association fibers in a U-shape, now a familiar anatomic design, connecting the temporal cortex with the lower region of the post central and pre-motor areas.^23^

Studies of lesions to this area reveal a disturbance called acoustic agnosia, or sensory aphasia (different from Wernicke's aphasia),^24^ and a deficiency in audio-verbal memory, in which the patient loses the ability to distinguish sounds of speech, specifically because sounds such as the ringing of objects are heard unaltered.^1^ Thus, it is important to note that ringing sounds are simple relative to speech sounds.

The impairment of audio-verbal memory produces disorders in the understanding of speech, naming of objects, recalling of words, and writing at the systemic cortical level which, in conjunction, cause the alienation of the meaning of words, resulting in speech disorder.^3^ These kinds of problems are not found in the right temporal lobe, in which symptoms of lesions were scarce, at least during Luria's era.

Lesions of the left middle temporal gyrus are associated with the loss of memory of series of sounds, syllables or words, as well as other defects causing a loss of reasoning, including disturbance in some intellectual operations.^3^^,^^25^ Interestingly, lesions to the left temporal lobe do not lead to problems associated with musical hearing or composition, unlike lesions to the right.^1^

**Figure 3:**
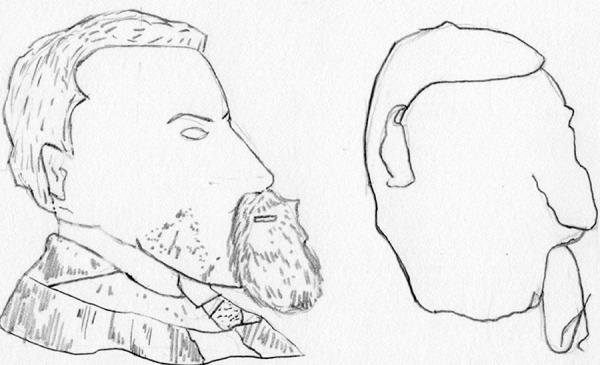
Pencil scheme from Luria (1973) of the drawings produced by a patient with the inability to draw a picture of a named object. A. Drawing done with patient looking at a model. B. Drawing done by the same patient without looking at a model - drawn only from memory.

Lesions of the posterior region of the temporal lobe close to the occipital lobe promote disturbances in the naming function of speech and an inability to evoke visual images after a word has been cited, for example the inability to draw a picture of a named object^1^^,^^18^ (Figure 3).

### More recent data on the neuropsychology of the temporal lobe

Luria and modern scientists used studies of lesions and pathologies of the cerebral organs as the basis for the development of neuropsychology.^4^ However, nowadays, it is no longer necessary to rely on post-mortem analysis alone, thanks to the emergence of new imaging techniques^3^ that also permit the study of the cerebral lobes *in situ,*^4^ which corroborates studied data on the function of the temporal lobe in normal speech.^26^

Furthermore, modern surgical techniques allow the study of more patients that survive after these procedures due to lower post-surgical mortality rates compared to those of surgical procedures utilizing older techniques. In some cases, the electroencephalography interictal can be used to detect neuropsychological deficiencies^27^ and a non-invasive procedure can also be performed using gamma knife radiosurgery.^28^

Modern studies include epilepsy as a neuropsychological problem of the temporal lobe in many more studies than during Luria's time, providing more detailed data on the hippocampus.^4^^,^^29^^-^34 In fact, epilepsy is the most studied subject in neuropsychology today (Figure 1).

These new subjects on the neuropsychology of the temporal lobe, i.e., epilepsy and the hippocampus, represent the main differences in relation to Luria's studies on this cerebral area.

In fact, neuropsychological data on lesions in the anterior part of the temporal lobe considering the hippocampus, obtained by functional magnetic resonance imaging analysis after surgical resection, revealed that, in cases of epilepsy linked to the activation of the left hippocampus, responsible for word encoding, they are linked to verbal memory deficits, whereas for the right temporal lobe, epilepsy results in the decline of visual memory.^4^^,^^5^

In functional terms and neuropsychological aspects, the medial temporal lobes work to maintain novel information in the absence of perceptual stimulus.^35^ Therefore, the cited lesions in these regions demonstrate that they are linked to short-term and long-term memory.^36^

On the other hand, there is a hypothesis that verbal memory is fractioned by the mesial temporal region, while verbal and non-verbal memory are not totally lateralized in the temporal lobes.^35^

Specifically, regarding cases of epilepsy, nothing was cited directly in Luria's work, putatively because epilepsy was not a direct field of his study,^37^ he did however, extensively cite functions of the temporal lobe.

Brain lesions in epilepsy are associated with neuropsychological tests used to assess the possible risks of postsurgical cognitive decline and enable the evaluation of patients before and after the surgery in relation to cognition^38^ and memory.^39^ A clinical assessment could thus be used to verify the expectations of the patient's post-surgery.^40^

Recent neuropsychological tests on individuals with epilepsy take into account processes such as intellectual abilities, attention, visuospatial skills, language, executive function, and memory,^41^ i.e., aspects studied and standardized by Luria,^1^ on which he cited the need for conducting more detailed tests in neuropsychological studies.^11^

The analysis of the temporal lobe in pre and post-surgical patients demonstrated a heterogeneous variation in relation to the neuropsychological tests used,^42^ for instance, Visual Naming, Logical Memory-Delayed, Logical Memory-Immediate, Learning Test,^41^^,^^43^ all cases not thoroughly discussed by Luria.

In this context, an earlier study suggested that, in intentional encoding, words activate the left temporal medial lobe, objects activate both medial temporal lobes symmetrically, and faces activate the right medial temporal lobe.^44^ In terms of the reorganization of functions in cases of cortical lesions, in patients with medial temporal lobe impairment, autobiographical memory became independent of this lobe.^45^

In patients with epilepsy, evaluations employing neuropsychological methods show that the decline in cognition was greater in the left temporal lobe than the right, in association with depression, a psychiatric comorbidity undertreated in cases of epilepsy,^46^ a fact not cited by Luria. In fact, as Luria did not work directly with epilepsy, it is, at least, improbable that he would have observed the association between epilepsy and depression. However, Luria's concepts indicate the basic processes associated with many neuropathologies,^38^ including that which serves as a basis in the analysis of new subjects.

The right and left temporal lobes seem to be associated with the perception of scary music, because the lesions in these regions diminish or deplete the patients' capacity to recognize aggressive songs.^47^ Regardless of Luria's studies on the effects of sounds and music on the temporal lobe, the different aspects of the kind of music language were not verified, but he did cite the theory of both hemispheres working together in many processes.^1^^,^^12^

Luria's studies cited the presence of cellular structures forming the cortical architecture using histological, physiological and anatomical data such as cortical layers, fascicles and Brodmann areas.^1^ Today, pathological problems are mainly associated with the cerebral networks discerned through neuropsychological tests, particularly those used for memory in the temporal lobe, with demonstrations on the reorganization of language involving increased pre-frontal activation.^42^

The general reorganization of the cortex, specifically in connection with temporal lobe epileptic surgery, is more promising in children and adolescents, putatively due to the higher probability of plasticity in younger people.^48^ However, this concept did not exist during Luria's time, but it is disclosed by neuropsychological tests. Nevertheless, in Luria's studies such as “The Man with a Shattered World: The History of a Brain Wound”,^2^ he demonstrated an attempt to stimulate other cerebral areas in order to compensate for the lesioned areas in the patient, perhaps a possible intuitive understanding of neural plasticity.

Thus, epileptic children have more compromised performance on neurophysiological tests, a decrease in brain tissue, mainly white-matter,^49^ and a high prevalence of intellectual dysfunction, suggesting a critical period for treatment in attempts to improve future cognition,^50^ considering neural plasticity.

According to Luria's theories, after a cortical lesion in the brain, the brain can compensate by distributing the specific function to other areas,^1^^,^^2^ particularly in children,^11^ as observed elsewhere.^42^ Potentially corroborating data may be, for example, which type of surgery for temporal lobe epilepsy improves neuropsychological test scores for the same patients, particularly those experiencing a reduction in seizures.^51^

Resection of part of the left temporal lobe generates memory loss, specifically verbal memory^34^ which increases with aging^52^ and causes impairment of language^53^ in part of, or for the remainder of, the patient's life time,^43^^,^^54^ an effect also found in lesions of the uncinate fasciculus, related to memory loss.^55^

Loss of semantic memory is a result of the effects of atrophy of the anterior temporal lobe, mainly the left side,^56^ as language impairment is associated with the region of the collateral sulcus and the anterior fusiform gyrus,^57^ part of the semantic system,^58^ not with the ventromedial frontal cortex.^59^ However, it seems to be a more extensive area encompassing a more extensive network.^58^^,^^60^ Memory problems with lesions in the temporal lobe, and the association with superior activities of the brain in the pre-frontal area, were extensively cited by Luria, albeit not necessarily with all of the frontal cortex.^2^ Indeed, latest results increasing the semantic area were not foreseen by Luria.

Thus, cortical restructuring occurs, but is incomplete or ineffective according to Luria's early observations, consistent with current observations,^61^ proposing that problems with left temporal lobe epilepsy could promote prefrontal metabolic asymmetry.^62^ Moreover, Luria cited detailed localization of language impairment in the temporal lobe in the secondary region of the temporal cortex.

Left temporal lobe epilepsy lesions, a regularly occurring even in epilepsy disorder^63^ and the most common type of medical intractable partial epilepsy,^56^ on top of hippocampal sclerosis, leads to verbal memory impairment in patients.^64^^-^66 Nevertheless, FMRI activation of the ipsilateral hippocampus in left temporal lobe epilepsy patients correlates with better memory when compared with contralateral hippocampus activation.^67^ In fact, there are data suggesting that hippocampal damage is the primary cause of deficiencies in non-verbal memory.^5^ Thus, both medial temporal lobes seem to be responsible for linking different cortical regions of the representation of memory.^68^ In fact, lesions in these regions cause amnesia.^69^^,^^70^

These results can also be found in amygdalohippocampectomy of patients with temporal lobe epilepsy.^71^ However, it is important to bear in mind that the lateralization of these functions was found in adolescents and adults,^72^ as well as pre-adolescents;^73^ and the hypothesis of the lateralization of the anterior temporal lobe was verified in relation to facial recognition, naming of the same entity^74^ and memory deficiencies.^29^

Studies on the effects of temporal lobe lesions and hippocampus atrophy^75^ caused by Alzheimer's show a strong correlation between mini-mental and memory tests, indicating an important means of diagnosing Alzheimer's.^76^ For providing a qualitative rating of medial temporal lobe atrophy, it is a fairly good diagnostic tool.^77^

In conjunction, the amygdala and hippocampus are associated with verbal intelligence deficiencies and increased anxiety and depression.^78^

Beyond the common problems associated with the temporal lobe and hippocampus, the reduction of gray matter in the left temporal lobe, together with reduced volume of the posterior superior temporal gyrus, is associated with schizophrenia, thought disorganization^79^ and episodes of aggression.^80^ On the other hand, radiotherapy generating temporal lobe necrosis showed problems in patients with memory, language, motor abilities and executive functions, but not in general intelligence.^6^ However, intellectual deficiencies are attributed to temporal lobe epilepsy.^32^

Nevertheless, mesial temporal lobe epilepsy and hippocampal sclerosis cause intellectual impairment as a non-specific symptom.^81^ Notwithstanding, there are reports that surgical treatment of temporal lobe epilepsy is ineffective, according to neuropsychological tests for verbal and visual memory, at least for small samples.^40^

These are some examples from Luria's studies supporting imaging tests of a disease that were not directly studied by him in the “The Working Brain”.^1^

Interestingly, bilateral disturbances in the temporal lobe could be associated with hypersexuality,^82^ data not cited by Luria.

## CONCLUSIONS

According to the data studied, Luria's epistemological foundation remains the basis for neuropsychological studies, but new data on the temporal lobe in relation to epilepsy and hippocampus analysis have been introduced into the scope of neurophysiology. In fact, the neuropsychological tests are used extensively in patients with temporal lobe epilepsy, before and after surgery, to verify cerebral activities. Nevertheless, there is some discrepancy according to some authors in relation to results, the asymmetry of functions, and the effects of temporal lobe surgery.

This investigation focused on earlier data from Luria's studies on the neurophysiological functions of the temporal lobe, comparing these with more recent data. However, in order to improve clinical aspects, a detailed study on the neuropsychological tests used for the temporal lobe should be performed.
